# The influence of MTHFR genetic polymorphisms on methotrexate therapy in pediatric acute lymphoblastic leukemia

**DOI:** 10.1515/biol-2021-0121

**Published:** 2021-11-06

**Authors:** Yaqing Shen, Zhujun Wang, Fen Zhou, Runming Jin

**Affiliations:** Department of Pediatrics, Union Hospital, Tongji Medical College, Huazhong University of Science and Technology, Wuhan, Hubei, 430022, China

**Keywords:** single nucleotide polymorphism, CCCG-ALL-2015, anticancer drug, drug toxicity

## Abstract

MTHFR is a crucial enzyme in folate metabolism. This study aimed to determine the relationship between MTHFR genetic polymorphism and elimination and toxicities of methotrexate (MTX). To do that, the study enrolled 145 patients diagnosed with acute lymphoblastic leukemia, who received chemotherapy following the Chinese Children’s Cancer Group Acute Lymphoblastic Leukemia (CCCG-ALL)-2015 protocol (clinical trial number: ChiCTR-IPR-14005706). We analyzed the effects of MTHFR C677T and A1298C polymorphisms on MTX elimination and toxicities. Patients with the MTHFR C677T TT genotype could tolerate a significantly higher MTX dose than those with the CC/CT genotype. However, patients with C677T TT genotypes had an increased risk of hypokalemia (1.369 to CC and 1.409 to CT types). The MTX infusion rate in patients with the MTHFR A1298C AC genotype was slightly lower than that in those with CC or AA genotypes. Patients with the A1298C AA genotype had a 1.405-fold higher risk of hepatotoxicity than those with the AC genotype (*P* > 0.05). There was no significant difference between the prevalence of other toxicities among MTHFR C677T or A1298C genotypes (*P* > 0.05). Neither MTHFR C677T nor A1298C polymorphisms were significantly associated with delayed MTX clearance. To conclude, MTHFR polymorphisms were not good predictors of MTX-related toxicities.

## 1 Introduction

**Figure 1:**
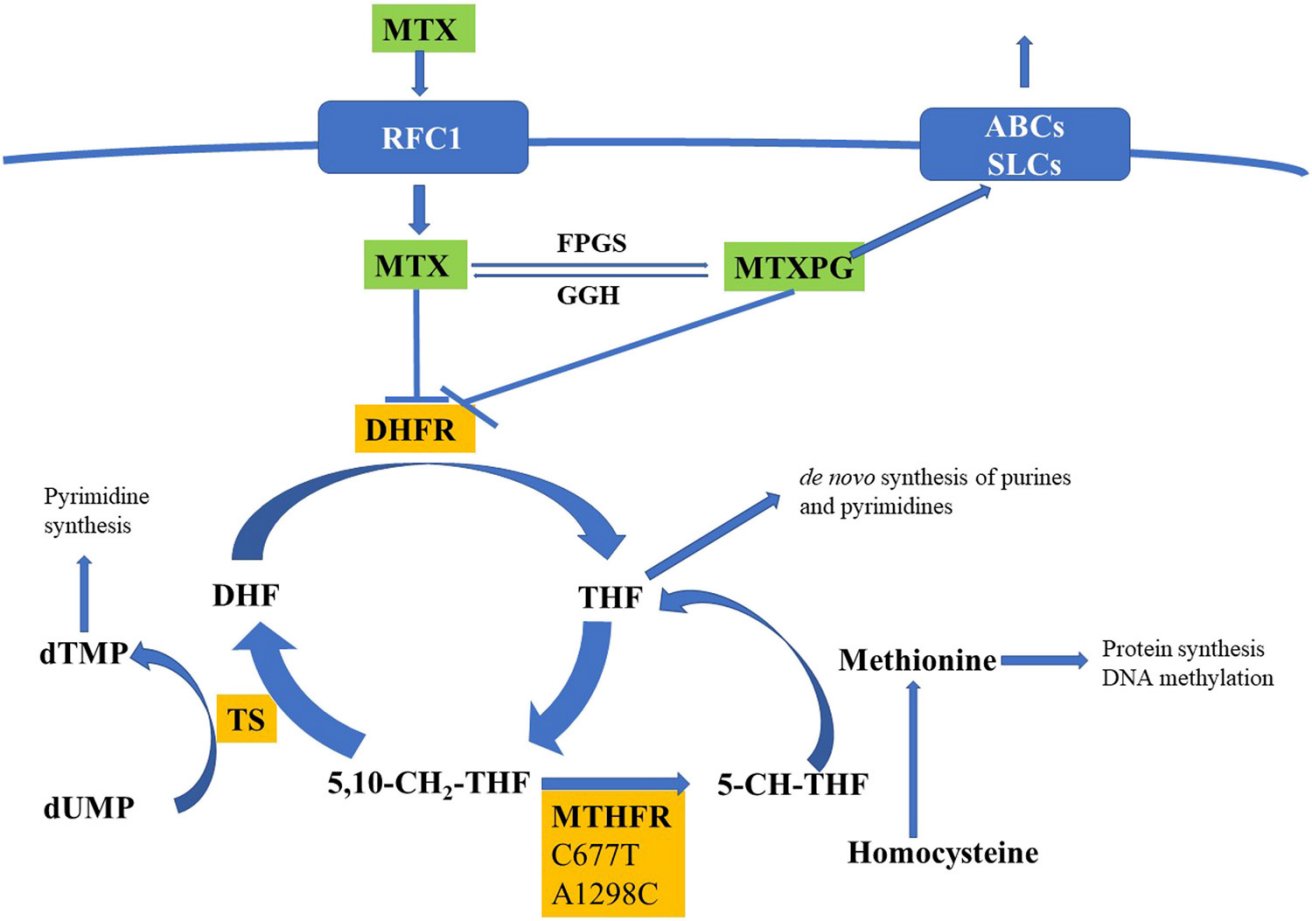
Simplified scheme of folate metabolism pathway targets of MTX. MTX competitively inhibits DHFR after entering cells through the RFC1. Abbreviations: MTX, methotrexate; MTXPG, methotrexate polyglutamated forms; RFC1, reduced folate carrier; ABCs, ABC family transporters; SLCs, SLC family transporters; DHF, dihydrofolate; THF, tetrahydrofolate; DHFR, dihydrofolate reductase; MTHFR, methylenetetrahydrofolate reductase; dTMP, deoxythymidine monophosphate; dUMP, deoxyuridine monophosphate; TS, Thymidylate synthase; 5-CH-THF, 5-methyltetrahydrofolate; 5,10-CH_2_-THF, 5,10-methylenetetrahydrofolate.

Methotrexate (MTX) is a crucial agent in treating pediatric acute lymphoblastic leukemia (ALL). It interferes with folate metabolism [1,2]. Upon entering cells through the reduced folate carrier, MTX competitively inhibits dihydrofolate reductase, which leads to reduced conversion of dihydrofolate to tetrahydrofolate. A deficiency of tetrahydrofolate inhibits the synthesis of DNA, RNA, and protein and exerts the antitumor effect of MTX [2,3] (Figure 1).

High-dose methotrexate (HD-MTX) chemotherapy is defined as MTX >500 mg/m^2^ and is part of the regimen for pediatric ALL [1,2]. High-intensity chemotherapy can improve the overall prognosis of childhood ALL, but can also cause severe toxicities. Therefore, MTX-induced toxicities are a major cause of treatment interruption and lead to an enhanced risk of relapse and even death [4].

The large interindividual and intraindividual variability in MTX pharmacokinetics hampers the prediction of the toxicities caused by MTX treatment [4,5]. In recent years, several studies have suggested that single nucleotide polymorphisms (SNPs) of the key enzymes and transporters in folic-acid metabolism are associated with MTX metabolism and the toxicities caused by MTX [2,3,6].

Methylenetetrahydrofolate reductase (MTHFR) is a key enzyme of the folic-acid metabolic pathway: it reduces 5,10-methyltetrahydrofolate to 5-methyltetrahydrofolate. Under the catalysis of thymidylate synthetase, 5,10-methyltetrahydrofolate changes dUMP to dTMP and participates in DNA synthesis. 5-methyltetrahydrofolate is a single-carbon provider involved in a wide range of methylation reactions *in vivo* [4,7] (shown in Figure 1). MTX cannot inhibit MTHFR directly, but changes in MTHFR activity affect intracellular folate distribution and methylation of nucleic acids, which may influence the efficacy and toxicities of MTX [4,8,9].

Two genetic polymorphisms can reduce the enzymatic activity of MTHFR. The first genetic polymorphism is rs1801133 (C677T), where the cytosine (C) of the 677th nucleotide mutates to thymine (T) and causes alanine to be replaced by valine [4]. Studies have suggested that the mutant CT genotype and TT genotype have 60 and 30% of enzymatic activity, respectively, compared with that of the wild genotype [10,11]. The other genetic polymorphism is rs1801131 (A1298C), where the adenine (A) of the 1298th nucleotide mutates to C and causes glutamic acid to be replaced by alanine [4]. It has been reported that patients with the CC genotype had 60–68% of enzymatic activity compared with that of the AA type [4,11]. Studies suggested that ALL patients with mutant types (CT/TT) of MTHFR C677T were more sensitive to HD-MTX chemotherapy and were at a higher risk of toxicities compared with individuals with the wild-type (CC) [12,13,14]. Additional studies have shown that the MTHFR A1298C AA genotype was associated with an increased risk of adverse events [4,15]. It has been suggested that the MTX dose should be reduced in patients with high-risk genotypes [6].

Based on previous research reports, MTHFR genetic polymorphism was recommended as one of the predictors of MTX-related toxicity in our hospital in recent years. However, in clinical practice, we found that MTHFR genetic polymorphism seems unlikely to accurately identify patients at risk from serious MTX-related toxicity. Therefore, in this pharmacogenetic study, we investigated the relationship between MTHFR C677T or MTHFR A1298C polymorphisms and the elimination and toxicities of MTX in Chinese children suffering from ALL. Herein, we aimed to determine the practicality of detecting MTHFR genetic polymorphisms.

## 2 Methods

### 2.1 Inclusion criteria and patients

The inclusion criteria were patients: (i) who are aged 0–14 years; (ii) who are diagnosed with ALL as the primary disease; (iii) who qualified for treatment according to the Chinese Children’s Cancer Group Acute Lymphoblastic Leukemia (CCCG-ALL)-2015 protocol (clinical trial number: ChiCTR-IPR-14005706); (iv) who were not suffering from another type of malignancy; (iv) who had completed at least one cycle of HD-MTX chemotherapy; (vi) from whom venous blood was collected and tested for MTHFR C677T and MTHFR A1298C polymorphisms; (vii) for whom the chemotherapy data and pharmacokinetic parameters of MTX were known.

A total of 145 patients from Union Hospital from November 2016 to September 2020 fulfilled the inclusion criteria and formed the study cohort.


**Informed consent:** Informed consent has been obtained from all the parents or guardians of children included in this study.
**Ethical approval:** The research related to human use has been complied with all the relevant national regulations, institutional policies, and in accordance with the tenets of the Helsinki Declaration and has been approved by the Human Research Ethics Committee of Union Hospital Affiliated to Tongji Medical College (Huazhong University of Science and Technology, Wuhan, China). Ethical permission number: S106-2015.

### 2.2 Treatment protocol of HD-MTX

Consolidation chemotherapy of CCCG-ALL-2015 consisted of four cycles of HD-MTX every 2 weeks and 6-mercaptopurine once a day given via the oral route. The recommended MTX dose was 3 g/m^2^ for low-risk (LR) patients and 5 g/m^2^ for intermediate/high-risk (IR/HR) patients. The MTX dose was reduced according to the creatinine clearance rate upon first exposure to HD-MTX and then adjusted based on the previous MTX concentration at 44 h (*C*_44h_). Ten percent of the full MTX dose was transfused (i.v.) within 30 min, and the remaining amount was pumped continuously for 23.5 h. Patients underwent prehydration at 200 mL/m^2^/h for 2–4 h or 100 mL/m^2^/h for > 12 h before infusion and then hydration at 3,000 mL/m^2^/day for 3 days. Sodium bicarbonate (5%) was given at 100 mL/m^2^ for 3 days from the day of MTX chemotherapy to maintain urine pH between 7 and 8. Leucovorin rescue was initiated 42 h from the beginning of the MTX infusion at a basic dose of 15 mg/m^2^/6 h and adjusted according to the MTX concentration. The MTX concentration was monitored every 24 h until it reached < 0.2 µmol/L.

### 2.3 MTHFR genotyping

Venous blood was collected and tested for MTHFR C677T and A1298C polymorphisms before MTX injection. MTHFR genotyping was performed in the Department of Pharmacy, Union Hospital Affiliated to Tongji Medical College of Huazhong University of Science and Technology, using the PCR-RFLP technique.

### 2.4 Data collection and definitions

We prospectively collected demographic information, details of HD-MTX chemotherapy (actual dose of MTX, MTX concentration, leucovorin dose), MTHFR polymorphisms, and MTX-related toxicities (e.g., myelosuppression, hepatotoxicity, acute kidney injury, mucositis, neurotoxicity). Patients were followed from the day of diagnosis of ALL to Aug 2021. In order to eliminate the bias caused by MTX dose adjustment and different recommended MTX doses (3 g/m^2^ for LR patients and 5 g/m^2^ for IR/HR patients), “MTX infusion rate (=actual MTX dose/recommended dose)” was used instead of “actual MTX dose”. Myelosuppression was represented by the number of days with absolute neutrophil count (ANC) < 1.0 × 10^9^/L. “Delay of MTX elimination” was defined as *C*_44h_ > 1.0 µmol/L or *C*_68h_ ≥ 0.2 µmol/L. Other toxicities were assessed based on the National Cancer Institute Common Toxicity Criteria v5.0 [16].

### 2.5 Statistical analyses

Continuous data are described as the median and range or interquartile range. Categorical data are presented as n (%). The Hardy–Weinberg equilibrium was tested for the genetic balance of MTHFR using the chi-square test. Relationships between MTHFR polymorphisms and the pharmacokinetics or toxicity of MTX were analyzed by the chi-square test (categorical data) or nonparametric test (continuous data). The risk factors for toxicities and delay in MTX elimination were analyzed by binary logistic regression. Relapse-free survival (RFS) probabilities were estimated using the Kaplan–Meier method together with the Log-rank test. *P* < 0.05 was considered significant. Statistical analyses were undertaken using SPSS v25.0 (IBM, Armonk, NY, USA).

## 3 Results

### 3.1 Demographic data of patients and genotype distribution

Of the 145 participants (88 male and 57 female) who formed our study, 130 had B-ALL, 15 had T-ALL; 52 were in the LR group; and 93 were in the IR/HR group. The median age was 5.0 years (range, 0.4–14.0 years). They received 576 cycles of HD-MTX chemotherapy.

For MTHFR C677T, the wild-type (CC) was the most common one, accounting for 40.0%, and the heterozygous mutant (CT) and homozygous mutant (TT) accounted for 36.6 and 23.4%, respectively. In the case of MTHFR A1298C, 65.5, 29.0, and 5.5% of patients had wild-type (AA), heterozygous mutant (AC), and homozygous mutant (CC), respectively. The Hardy–Weinberg equilibrium test indicated that the gene frequency in the sample population was in accordance with the genetic balance law and that the sample population was representative (P > 0.05) (Table 1).

**Table 1:** Relationship between MTHFR polymorphisms and MTX chemotherapy or MTX concentrations

Characteristic	C677T			*P*	A1298C			*P*
	CC	CT	TT		AA	AC	CC	
N (%)	58 (40.0%)	53 (36.6%)	34 (23.4%)	0.75^*^	95 (65.5%)	42 (29.0%)	8 (5.5%)	0.99^*^
Age (years)	5.2 (3.4–8.0)	4.3 (3.0–9.0)	6.0 (4.0–9.1)	0.412	6.0 (3.7–9.1)	4.5 (3.0–7.2)	4.3 (1.5–7.0)	0.145
Sex				0.101				0.279
Male	41	27	20		56	25	7	
Female	17	26	14		39	17	1	
Risk group				0.936				0.653
Low	20	19	13		33	17	2	
Intermediate/high	38	34	21		62	25	6	
MTX infusion rate	0.96 (0.7–1.0)	1.0 (0.7–1.0)	1.0 (0.8–1.0)	0.026	1.0 (0.8–1.0)	0.85 (0.7–1.0)	1.0 (0.8–1.0)	0.008
Leucovorin dose (mg/m^2^)	75 (75–135)	75 (75–195)	75 (75–221)	0.22	75 (75–195)	75 (75–146)	75 (75–75)	0.057
*C*_44h_ (μmol/L)	0.61 (0.33–3.38)	0.74 (0.47–1.95)	0.7 (0.37–2.91)	0.822	0.58 (0.39–1.22)	0.51 (0.36–1.01)	0.51 (0.42–0.82)	0.266
≤1.0	37 (63.8%)	31 (58.5%)	20 (58.8%)		57 (60.0%)	24 (57.1%)	7 (87.5%)	
>1.0	21 (36.2%)	22 (41.5%)	14 (41.2%)		38 (40.0%)	18 (42.9%)	1 (12.5%)	
*C*_68h_ (μmol/L)	0.2 (0.1–0.54)	0.21 (0.14–0.55)	0.23 (0.1–0.61)	0.663	0.19 (0.11–0.35)	0.16 (0.10–0.30)	0.18 (0.1–0.25)	0.351
<0.2	30 (51.7%)	23 (43.4%)	17 (50.0%)		45 (47.4%)	20 (47.6%)	5 (62.5%)	
≥0.2	28 (48.3%)	30 (56.6%)	17 (50.0%)		50 (52.6%)	22 (52.4%)	3 (37.5%)	

^*^Hardy–Weinberg test.

*C*_44h_, MTX concentration at 44 h; *C*_68h_, MTX concentration at 68 h.

### 3.2 MTX chemotherapy and MTX elimination

There was no significant difference in the distribution of sex, age, immunotyping, or risk group among all the MTHFR C677T and MTHFR A1298C genotypes (*P* > 0.05) (Table 1). For MTX chemotherapy and MTX elimination, only the MTX infusion rate (actual MTX dose/recommended dose) was significantly associated with MTHFR polymorphisms (*P* < 0.05). The median MTX infusion rate of MTHFR C677T CC/CT/TT genotypes was 0.96 (IQR, 0.7–1.0), 1.0 (IQR, 0.7–1.0), and 1.0 (IQR, 0.8–1.0), respectively. The subsequent pairwise nonparametric test showed that the MTX infusion rate for the TT genotype was significantly higher than that of CC and CT genotypes (*P* = 0.011 and *P* = 0.019), whereas there was no significant difference between the CC and CT genotypes (*P* = 0.838).

With regard to MTHFR A1298C, the median MTX infusion rate of the AA/AC/CC genotypes was 1.0 (IQR, 0.8–1.0), 0.85 (IQR, 0.7–1.0), and 1.0 (IQR, 0.8–1.0), respectively. The subsequent pairwise nonparametric test showed that patients with the AC genotype had a higher MTX infusion rate than that of patients with the AA genotype or CC genotype (*P*_AC/AA_ < 0.001, *P*_AC/CC_ = 0.001, *P*_AA/CC_ = 0.111).

Delayed elimination of MTX at 44 and 68 h was noted in 39.3 and 51.7% of cycles, respectively. Neither MTHFR C677T nor MTHFR A1298C polymorphisms were associated with the leucovorin dose or delay of MTX clearance when evaluated by the chi-square test or nonparametric test.

### 3.3 HD-MTX-induced toxicities

MTX-related toxicities were noted in 80.2% (461/576) cycles and were mainly of grade 1/2. Grade-3/4 toxicity was found in 18.6% (107/576) cycles, of which infection accounted for 60.7%. Myelosuppression was the most common complication, followed by hepatotoxicity (Table 2). Further, 20.1% of cycles had ANC ≤ 1.0 × 10^9^/L lasting >7 days, whereas hepatotoxicity occurred in 37.1% of cycles (5.2% of which were graded 3/4).

**Table 2:** MTX-related toxicities

Toxicity	*n*	%
Myelosuppression		
1–7 days	244	42.4
>7 days	116	20.1
Length of chemotherapy delay		
1–7 days	17	3.0
>7 days	8	1.4
Hepatotoxicity grade		
1/2	184	31.9
3/4	30	5.2
Hypokalemia	89	15.4
Hyperkalemia	12	2.1
Grade of gastrointestinal toxicity		
1/2	32	5.6
3/4	9	1.6
Infection (grade 3/4)	65	11.3
Nephrotoxicity grade		
1/2	4	0.7
3/4	5	0.9
Mucositis grade		
1/2	26	4.5
3/4	7	1.2
Neurotoxicity grade		
1/2	1	0.2
3/4	9	1.6

### 3.4 Risk factors for grade-3/4 toxicities and delay in MTX elimination

Predictors with a significant difference in the univariate analysis or identified clinically were added in the binary logistic regression model. Nine predictors were included (Table 3). Being female, IR/HR, and *C*_44h_ > 1.0 μmol/L were risk factors for grade-3/4 toxicity (*P* < 0.05). Age ≥5 years, male sex, IR/HR, and MTX infusion rate ≥0.8 were risk factors for delayed elimination of MTX. Neither MTHFR C677T nor MTHFR A1298C was associated with the prevalence of severe adverse events or MTX elimination (*P* > 0.05).

**Table 3:** Risk factors for grade-3/4 toxicities and delay in MTX elimination according to binary logistic regression

Characteristic*	Toxicity^**^	*C*_44h_	*C*_68h_
	P/OR	P/OR	P/OR
Age (<5 vs ≥5 years)	0.781/0.936	0.021/1.616	0.009/1.638
Sex (female vs male)	0.016/0.558	0.005/1.834	0.002/1.832
Immunophenotype (B-ALL vs T-ALL)	0.766/0.895	0.573/0.837	0.544/1.202
Risk group (LR vs IR/HR)	0.045/1.727	0.000/3.014	0.000/2.324
MTX infusion rate (<0.8 vs ≥0.8)	0.318/1.274	0.001/2.059	0.000/1.993
*C*_44h_ (≤1.0 vs >1.0 μmol/L）	0.002/3.017	—	—
*C*_68h_ (<0.2 vs ≥0.2 μmol/L)	0.900/1.045	—	—
C677T			
CC	0.599	0.087	0.118
CT	0.833/0.944	0.057/1.367	0.095/1.255
TT	0.328/0.731	0.164/1.480	0.261/1.334
A1298C			
AA	0.177	0.586	0.925
AC	0.191/0.693	0.976/1.007	0.737/1.076
CC	0.300/1.639	0.312/0.602	0.892/0.945

^*^The first entry is the reference group.

^**^Grade-3/4 toxicities.

*C*_44h_: MTX concentration at 44 h; *C*_68h_, MTX concentration at 68 h.

### 3.5 Relationship between MTHFR polymorphisms and MTX-related toxicity

The chi-square test was used to detect the difference in the prevalence of MTX-related toxicities among the genotypes tested (Table 4). The MTHFR C677T polymorphism was found to be significantly associated with the prevalence of hypokalemia. Patients with the TT genotype were at a higher risk of hypokalemia than those with the CC type or CT type (*P*_TT/CC_ = 0.008, OR_TT/CC_ = 1.369, 95% CI: 1.045–1.792; *P*_TT/CT_ = 0.007, OR_TT/CT_ = 1.409, 95% CI: 1.053–1.884), whereas there was no significant difference between patients with the CC genotype or CT genotype (*P* = 0.923). None of the other toxicities were significantly associated with the MTHFR C677T polymorphism (*P* > 0.05).

**Table 4:** Relationship between MTHFR polymorphisms and MTX-related toxicity

Toxicity	C677T			*P*	A1298C			*P*
	CC (*n* = 230)	CT (*n* = 212)	TT (*n* = 134)		AA (*n* = 376)	AC (*n* = 168)	CC (*n* = 32)	
Myelosuppression				0.623				0.230
1–7 days	103	86	55		158	69	17	
>7 days	43	49	24		68	41	7	
Chemotherapy delay (days)	11	12	2	0.258	14	8	3	0.306
Hepatotoxicity	93	71	50	0.564	153	50	11	0.048^***^
Hypokalemia	29	27	32	0.009^**^	66	19	4	0.158
Hyperkalemia	8	3	1	0.147	7	3	2	0.236
Gastrointestinal toxicity	18	11	12	0.358	30	11	0	0.228
Infection	26	25	15	0.178	44	15	7	0.105
Nephrotoxicity	4	1	4	0.428	7	2	0	0.645
Mucositis	9	14	10	0.294	20	11	2	0.843
Neurotoxicity	3	7	0	0.059	7	3	0	0.740

^*^Including toxicities graded 1/2/3/4.

^**^ *P*_TT/CC_ = 0.008, OR_TT/CC_ = 1.369, 95% CI: 1.045–1.792; *P*_TT/CT_ = 0.007, OR_TT/CT_ = 1.409, 95% CI: 1.053–1.884.

^***^ *P*_AA/AC_ = 0.015, OR_AA/AC_ = 1.405, 95% CI: 1.060–1.862.

The chi-square test suggested that the MTHFR A1298C polymorphism was associated with an increased risk of hepatotoxicity (*P* = 0.048). Patients with the AA genotype had a 1.405-fold higher risk of hepatotoxicity than those with the AC genotype. There was no significant relationship between the MTHFR A1298C polymorphism and myelosuppression, chemotherapy delay, neurotoxicity, hyperkalemia, hypokalemia, infection, mucositis, or nephrotoxicity (*P* > 0.05).

### 3.6 MTHFR polymorphisms with treatment outcome

**Figure 2:**
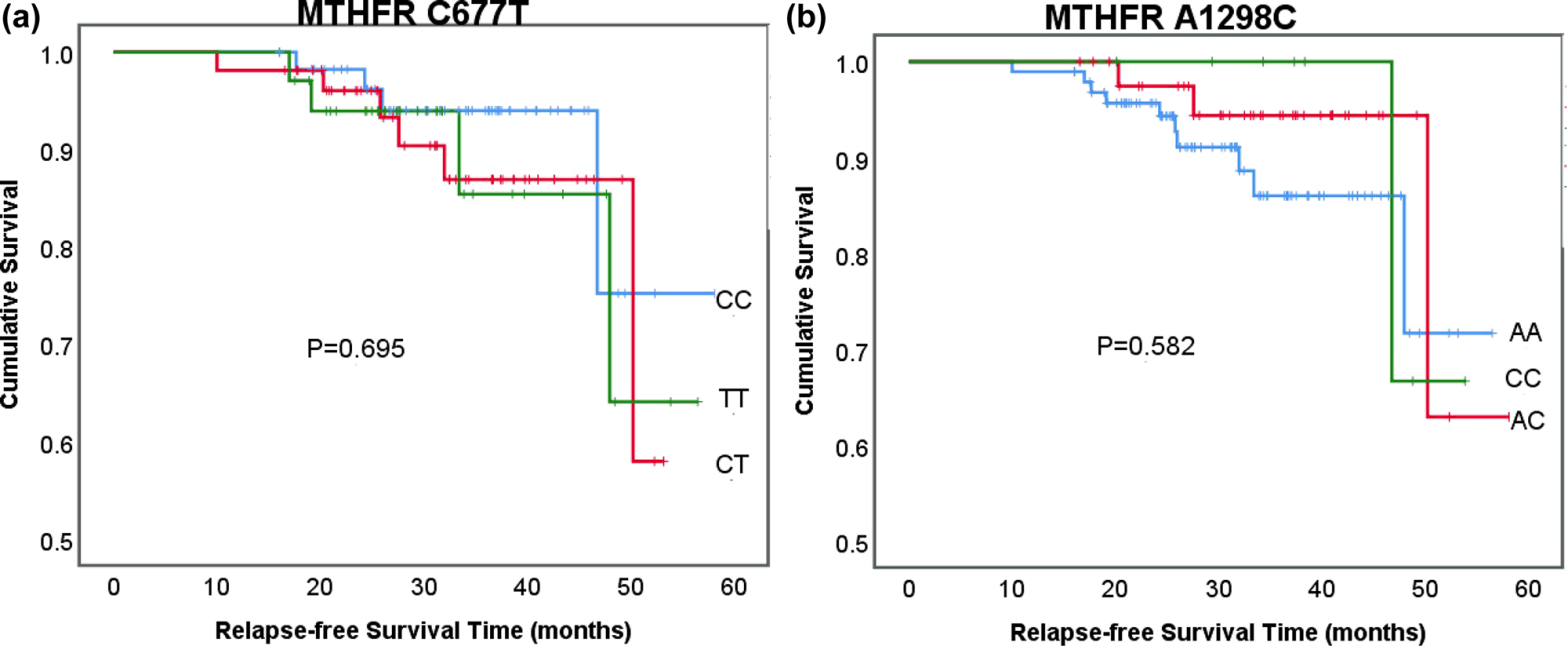
MTHFR polymorphisms and relapse-free survival. (a) MTHFR C677T and (b) MTHFR A1298C.

As of Aug 2021, the median follow-up time was 31.3 months (range: 10–58). One hundred and seventeen patients survived without events, 14 patients relapsed (1 in the testes, 9 in bone marrow, 1 in central nervous system, 3 combined), 11 patients were lost to follow-up, 1 patient abandoned because of serious adverse events, and 2 patients died. No one died from HD-MTX treatment. To detect the influence of MTHFR polymorphisms on treatment outcomes of pediatric ALL patients, we did both univariate and multivariate analyses. No significant correlation between MTHFR C677T or A1298C and RFS was found. The survival curves drawn by the Kaplan-Meier method are shown in Figure 2.

## 4 Discussion

MTX is a crucial therapeutic agent for the successful treatment of ALL [1]. Since its first clinical trial in 1953 using a mini-dosage, the overall prognosis of childhood ALL has ameliorated [1]. Higher MTX dose increases the cure rate of childhood ALL [17]. However, intensive chemotherapy will increase the incidence of MTX-related toxicity, leading to chemotherapy delay, long-term sequelae, or even death [6]. Even with adequate hydration and aggressive monitoring of MTX concentration when receiving HD-MTX chemotherapy, some patients will experience severe adverse events [6]. It is still a major clinical challenge to identify a predictor of the occurrence of MTX-related toxicity. MTHFR genetic polymorphism is one of the predictors studied. However, the conclusions drawn have been controversial.

Our prospective study enrolled 145 children diagnosed with ALL and treated following the CCCG-ALL-2015 protocol in China. We wished to investigate whether the MTHFR C677T and MTHFR A1298C polymorphisms were correlated with MTX elimination or MTX-related toxicities in the Chinese group.

In the present study, the rate of homozygous mutation of MTHFR C677T and MTHFR A1298C was 23.4 and 5%, whereas the heterozygous mutation rate was 36.6 and 29.0%, respectively. The Hardy–Weinberg equilibrium test indicated that the gene frequency in the sample population was in accordance with the genetic balance law and that the sample population was representative.

In our research, although the C677T polymorphism was not associated directly with a delay in MTX clearance, patients with the TT genotype had a higher MTX infusion rate. This finding suggested that they could tolerate a higher MTX dose and were less likely to experience dose reduction, which was based on a high *C*_44h_ previously. This result was similar to data documented by Cwiklinska and colleagues [12], which suggested that patients with the TT genotype achieved significantly lower steady state MTX concentrations. However, Chae et al. [18] postulated that, in patients with the C677T genotype, the MTX dose was adjusted more frequently than in those with CC or CT genotypes. Faganel and coworkers [19] indicated that the MTX clearance in individuals with the TT genotype decreased to 73.8%. Suthandiram et al. [20], Yanagimachi and colleagues [21], EL-Khodary and coworkers [22], Kantar and collaborators [23], and Esmaili et al. [24] came to similar conclusions. Studies have shown that MTHFR was not associated with MTX clearance [14,25,26,27].

Apart from the observation that patients with the TT genotype were at a higher risk of hypokalemia, there was no significant relationship between the MTHFR C677T polymorphism and the prevalence of other MTX-related toxicities. To date, research on the relationship between hypokalemia and MTHFR polymorphisms has not been published. MTX is mainly eliminated by renal excretion. MTX and its metabolites can precipitate and crystallize in renal tubules, leading to acute kidney injury [6]. This may be the cause of abnormal serum potassium. Moreover, insufficient volume and urine acidity are major risk factors for acute kidney injury, so hydration and urine alkalinity are necessary to mitigate kidney damage during HDMTX therapy [6]. Increased potassium excretion due to rapid hydration may also be the cause of hypokalemia. MTX dose is another risk factor for nephrotoxicity [1]. In our research, the results suggested that patients with the TT genotype received a higher MTX dose. Therefore, we hypothesized that although there was no significant relationship between the TT genotype and the prevalence of nephrotoxicity in our study, it would ultimately lead to increased nephrotoxicity and affect potassium metabolism.

Whether the MTHFR C677T polymorphism is related to toxicities is controversial. However, studies have suggested that ALL patients with MTHFR C677T mutant types (CT/TT) were more sensitive to HD-MTX chemotherapy and at a higher risk of toxicities compared with individuals with the wild-type mutant (CC) [12,13,14,22,28,29]. For instance, a prospective study involving 74 cases indicated that the T allele was associated with an increased risk of hematopoietic toxicity. Other studies discovered that mucositis, diarrhea, anemia, hepatotoxicity, or leukemia were more frequent in patients with the mutant genotype than in those with the wild-type [18,19,25,30,31,32]. However, the types of toxicities related to the MTHFR C677T polymorphism differed in those studies. Moreover, some studies found no relationship between MTHFR C677T- and MTX-related toxicities or drew the opposite conclusion [23,33,34,35].

With regard to the MTHFR A1298C polymorphism, we noticed that patients with the AA genotype carried a 1.405-fold higher risk of hepatotoxicity than those with the AC genotype. There was no relationship between the MTHFR polymorphism and myelosuppression, chemotherapy delay, neurotoxicity, hyperkalemia, hypokalemia, infection, mucositis, or nephrotoxicity. The delay in MTX elimination was irrelevant to the MTHFR A1298C gene polymorphism. It has been reported that the C allele was a protective factor of MTX-related toxicities (e.g., hepatotoxicity, neutropenia, mucositis) [19,24,27]. However, studies conducted by Yousef et al. [25], Moulik and colleagues [32], and Eissa and Ahmed [27] suggested that the MTHFR A1298C CC genotype was associated with an increased risk of cytopenia. Other studies concluded that MTHFR A1298C was not related to MTX-related toxicity or delay in MTX elimination [20,30,34,36].

The controversies mentioned above may have been caused by various confounding factors in the research process: population, chemotherapy protocol, sample size, toxicity-assessment method, as well as large interindividual and intraindividual variability in MTX pharmacokinetics [5]. Several factors can affect MTX clearance and the prevalence of MTX-related toxicities, such as age, sex, and MTX dose, as reported in our study. Renal function, amount of hydration, urine pH, dose, and timing of leucovorin administration have also been reported to be the main factors in MTX-related toxicities and a delay in MTX clearance [6]. Therefore, MTHFR polymorphisms are not the main factor leading to toxicity or delay in MTX clearance in the Chinese group, although there may be some relationship between them.

Since altered MTX pharmacokinetics may affect treatment efficacy, we investigated the effect of MTHFR polymorphisms on patients’ RFS. However, there was no significant correlation between MTHFR C677T or A1298C and RFS. He et al. [37] published a systematic review including six studies on the relationship between MTHFR polymorphisms and pediatric ALL outcome in 2014. Two studies showed that there was a higher relapse risk in individuals with the MTHFR 677TT genotype. Nevertheless, Umerez et al. [38] performed a new literature review from 2013 to 2016. Eight studies have analyzed the association between MTHFR C677T polymorphism and pediatric ALL outcome, but none showed a significant association. With regard to MTHFR A1298C, two studies showed significant results but with the opposite effect. All these findings support that MTHFR polymorphisms might not be good outcome predictors for childhood ALL.

## 5 Conclusion

There was some correlation between MTHFR C677T or MTHFR A1298C polymorphisms and MTX-related toxicities. However, MTHFR polymorphisms were not good predictors of toxicities or a delay in MTX clearance. The role of MTHFR polymorphisms in guiding consolidation chemotherapy is limited.

## References

[1] Wijaya J, Gose T, Schuetz JD. Using pharmacology to squeeze the life out of childhood leukemia, and potential strategies to achieve breakthroughs in medulloblastoma treatment. Pharmacol Rev. 2020;72(3):668–91. 10.1124/pr.118.016824 PMC7312347 32571983

[2] Bleyer WA. The clinical pharmacology of methotrexate: new applications of an old drug. Cancer. 1978;41(1):36–51. 10.1002/1097-0142(197801)41:1<36::aid-cncr2820410108>3.0.co;2-i 342086

[3] Giletti A, Esperon P. Genetic markers in methotrexate treatments. Pharmacogenomics J. 2018;18(6):689–703. 10.1038/s41397-018-0047-z 30237581

[4] Zahra FT, Nahid NA, Islam MR, Al-Mamun MMA, Apu MNH, Nahar Z, et al. Pharmacogenetic variants in MTHFR gene are significant predictors of methotrexate toxicities in Bangladeshi patients with acute lymphoblastic leukemia. Clin Lymphoma Myeloma Leuk. 2020;20(2):E58–65. 10.1016/j.clml.2019.11.020 31884153

[5] Evans WE, Crom WR, Abromowitch M, Dodge R, Look AT, Bowman WP, et al. Clinical pharmacodynamics of high-dose methotrexate in acute lymphocytic leukemia. Identification of a relation between concentration and effect. N Engl J Med. 1986;314(8):471–7. 10.1056/NEJM198602203140803 3456079

[6] Howard SC, McCormick J, Pui CH, Buddington RK, Harvey RD. Preventing and managing toxicities of high-dose methotrexate. Oncologist. 2016;21(12):1471–82. 10.1634/theoncologist.2015-0164 PMC5153332 27496039

[7] Koppen IJ, Hermans FJ, Kaspers GJ. Folate related gene polymorphisms and susceptibility to develop childhood acute lymphoblastic leukaemia. Br J Haematol. 2010;148(1):3–14. 10.1111/j.1365-2141.2009.07898.x 19775302

[8] Dulucq S, St-Onge G, Gagné V, Ansari M, Sinnett D, Labuda D, et al. DNA variants in the dihydrofolate reductase gene and outcome in childhood ALL. Blood. 2008;111(7):3692–700. 10.1182/blood-2007-09-110593 18096764

[9] de Jonge R, Hooijberg JH, van Zelst BD, Jansen G, van Zantwijk CH, Kaspers GJ, et al. Effect of polymorphisms in folate-related genes on in vitro methotrexate sensitivity in pediatric acute lymphoblastic leukemia. Blood. 2005;106(2):717–20. 10.1182/blood-2004-12-4941 15797993

[10] Schwahn B, Rozen R. Polymorphisms in the methylenetetrahydrofolate reductase gene: clinical consequences. Am J Pharmacogenomics. 2001;1(3):189–201. 10.2165/00129785-200101030-00004 12083967

[11] Weisberg I, Tran P, Christensen B, Sibani S, Rozen R. A second genetic polymorphism in methylenetetrahydrofolate reductase (MTHFR) associated with decreased enzyme activity. Mol Genet Metab. 1998;64(3):169–72. 10.1006/mgme.1998.2714 9719624

[12] Cwiklinska M, Czogala M, Kwiecinska K, Madetko-Talowska A, Szafarz M, Pawinska K, et al. Polymorphisms of SLC19A1 80 G > A, MTHFR 677 C > T, and Tandem TS repeats influence pharmacokinetics, acute liver toxicity, and vomiting in children with acute lymphoblastic leukemia treated with high doses of methotrexate. Front Pediatr. 2020;8:307. 10.3389/fped.2020.00307 PMC7308427 32612964

[13] Seidemann K, Book M, Zimmermann M, Meyer U, Welte K, Stanulla M, et al. MTHFR 677 (C → T) polymorphism is not relevant for prognosis or therapy-associated toxicity in pediatric NHL: results from 484 patients of multicenter trial NHL-BFM 95. Ann Hematol. 2006;85(5):291–300. 10.1007/s00277-005-0072-2 16463153

[14] Shimasaki N, Mori T, Samejinia R, Sato R, Shimada IK, Yahagi N, et al. Effects of methylenetetrahydrofolate reductase and reduced folate carrier 1 polymorphisms on high-dose methotrexate-induced toxicities in children with acute lymphoblastic leukemia or lymphoma. J Pediatr Hematol Oncol. 2006;28(2):64–8. 10.1097/01.mph.0000198269.61948.90 16462575

[15] Yao P, He X, Zhang R, Tong R, Xiao H. The influence of MTHFR genetic polymorphisms on adverse reactions after methotrexate in patients with hematological malignancies: a meta-analysis. Hematology. 2019;24(1):10–9. 10.1080/10245332.2018.1500750 30024839

[16] Institute NC, Institute NC. Common terminology criteria for adverse events (CTCAE) version 5.0. National Cancer Institute; 2017 Nov 27. Available from: https://ctep.cancer.gov/protocolDevelopment/electronic_applications/docs/CTCAE_v5_Quick_Reference_8.5x11.pdf.

[17] Abromowitch M, Ochs J, Pui CH, Kalwinsky D, Rivera GK, Fairclough D, et al. High-dose methotrexate improves clinical outcome in children with acute lymphoblastic leukemia: St. Jude total therapy study X. Med Pediatr Oncol. 1988;16(5):297–303. 10.1002/mpo.2950160502 3054451

[18] Chae H, Kim M, Choi SH, Kim SK, Lee JW, Chung NG, et al. Influence of plasma methotrexate level and MTHFR genotype in Korean paediatric patients with acute lymphoblastic leukaemia. J Chemother. 2020;32(5):251–9. 10.1080/1120009X.2020.1764280 32431230

[19] Faganel Kotnik B, Grabnar I, Bohanec Grabar P, Dolzan V, Jazbec J. Association of genetic polymorphism in the folate metabolic pathway with methotrexate pharmacokinetics and toxicity in childhood acute lymphoblastic leukaemia and malignant lymphoma. Eur J Clin Pharmacol. 2011;67(10):993–1006. 10.1007/s00228-011-1046-z 21509569

[20] Suthandiram S, Gan GG, Zain SM, Bee PC, Lian LH, Chang KM, et al. Effect of polymorphisms within methotrexate pathway genes on methotrexate toxicity and plasma levels in adults with hematological malignancies. Pharmacogenomics. 2014;15(11):1479–94. 10.2217/pgs.14.97 25303299

[21] Yanagimachi M, Goto H, Kaneko T, Naruto T, Sasaki K, Takeuchi M, et al. Influence of pre-hydration and pharmacogenetics on plasma methotrexate concentration and renal dysfunction following high-dose methotrexate therapy. Int J Hematol. 2013;98(6):702–7. 10.1007/s12185-013-1464-z 24241962

[22] EL-Khodary NM, EL-Haggar SM, Eid MA, Ebeid EN. Study of the pharmacokinetic and pharmacogenetic contribution to the toxicity of high-dose methotrexate in children with acute lymphoblastic leukemia. Med Oncol. 2012;29(3):2053–62. 10.1007/s12032-011-9997-6 21644011

[23] Kantar M, Kosova B, Cetingul N, Gumus S, Toroslu E, Zafer N, et al. Methylenetetrahydrofolate reductase C677T and A1298C gene polymorphisms and therapy-related toxicity in children treated for acute lymphoblastic leukemia and non-Hodgkin lymphoma. Leuk Lymphoma. 2009;50(6):912–7. 10.1080/10428190902893819 19391036

[24] Esmaili MA, Kazemi A, Faranoush M, Mellstedt H, Zaker F, Safa M, et al. Polymorphisms within methotrexate pathway genes: Relationship between plasma methotrexate levels, toxicity experienced and outcome in pediatric acute lymphoblastic leukemia. Iran J Basic Med Sci. 2020;23(6):800–9. 10.22038/ijbms.2020.41754.9858 PMC7351433 32695297

[25] Yousef AM, Farhad R, Alshamaseen D, Alsheikh A, Zawiah M, Kadi T. Folate pathway genetic polymorphisms modulate methotrexate-induced toxicity in childhood acute lymphoblastic leukemia. Cancer Chemother Pharmacol. 2019;83(4):755–62. 10.1007/s00280-019-03776-8 30684021

[26] Wang SM, Sun LL, Zeng WX, Wu WS, Zhang GL. Influence of genetic polymorphisms of FPGS, GGH, and MTHFR on serum methotrexate levels in Chinese children with acute lymphoblastic leukemia. Cancer Chemother Pharmacol. 2014;74(2):283–9. 10.1007/s00280-014-2507-8 24908438

[27] Eissa DS, Ahmed TM. C677T and A1298C polymorphisms of the methylenetetrahydrofolate reductase gene: effect on methotrexate-related toxicity in adult acute lymphoblastic leukaemia. Blood Coagul Fibrinolysis. 2013;24(2):181–8. 10.1097/MBC.0b013e32835b249d 23183238

[28] Yang L, Hu X, Xu L. Impact of methylenetetrahydrofolate reductase (MTHFR) polymorphisms on methotrexate-induced toxicities in acute lymphoblastic leukemia: a meta-analysis. Tumour Biol. 2012;33(5):1445–54. 10.1007/s13277-012-0395-2 22528943

[29] Ulrich CM, Yasui Y, Storb R, Schubert MM, Wagner JL, Bigler J, et al. Pharmacogenetics of methotrexate: toxicity among marrow transplantation patients varies with the methylenetetrahydrofolate reductase C677T polymorphism. Blood. 2001;98(1):231–4. 10.1182/blood.v98.1.231 11418485

[30] Zhu C, Liu YW, Wang SZ, Li XL, Nie XL, Yu XT, et al. Associations between the C677T and A1298C polymorphisms of MTHFR and the toxicity of methotrexate in childhood malignancies: a meta-analysis. Pharmacogenomics J. 2018;18(3):450–9. 10.1038/tpj.2017.34 28696419

[31] Mahmoud LB, Mdhaffar M, Frikha R, Ghozzi H, Hakim A, Sahnoun Z, et al. Use of MTHFR C677T polymorphism and plasma pharmacokinetics to predict methotrexate toxicity in patients with acute lymphoblastic leukemia. Adv Clin Exp Med. 2018;27(8):1061–8. 10.17219/acem/69802 29911750

[32] Roy Moulik N, Kumar A, Agrawal S, Awasthi S, Mahdi AA, Kumar A. Role of folate status and methylenetetrahydrofolate reductase genotype on the toxicity and outcome of induction chemotherapy in children with acute lymphoblastic leukemia. Leuk Lymphoma. 2015;56(5):1379–84. 10.3109/10428194.2014.947608 25065700

[33] Ramirez-Pacheco A, Moreno-Guerrero S, Alamillo I, Medina-Sanson A, Lopez B, Moreno-Galvan M. Mexican childhood acute lymphoblastic leukemia: a pilot study of the MDR1 and MTHFR gene polymorphisms and their associations with clinical outcomes. Genet Test Mol Biomarkers. 2016;20(10):597–602. 10.1089/gtmb.2015.0287 27533339

[34] Erculj N, Kotnik BF, Debeljak M, Jazbec J, Dolzan V. Influence of folate pathway polymorphisms on high-dose methotrexate-related toxicity and survival in childhood acute lymphoblastic leukemia. Leuk Lymphoma. 2012;53(6):1096–104. 10.3109/10428194.2011.639880 22074251

[35] Kishi S, Griener J, Cheng C, Das S, Cook EH, Pei D, et al. Homocysteine, pharmacogenetics, and neurotoxicity in children with leukemia. J Clin Oncol. 2003;21(16):3084–91. 10.1200/JCO.2003.07.056 12915598

[36] Lopez-Lopez E, Martin-Guerrero I, Ballesteros J, Garcia-Orad A. A systematic review and meta-analysis of MTHFR polymorphisms in methotrexate toxicity prediction in pediatric acute lymphoblastic leukemia. Pharmacogenomics J. 2013;13(6):498–506. 10.1038/tpj.2012.44 23089671

[37] He HR, Liu P, He GH, Dong WH, Wang MY, Dong YL, et al. Association between reduced folate carrier G80A polymorphism and methotrexate toxicity in childhood acute lymphoblastic leukemia: a meta-analysis. Leuk Lymphoma. 2014;55(12):2793–800. 10.3109/10428194.2014.898761 24597986

[38] Umerez M, Gutierrez-Camino Á, Muñoz-Maldonado C, Martin-Guerrero I, Garcia-Orad A. MTHFR polymorphisms in childhood acute lymphoblastic leukemia: influence on methotrexate therapy. Pharmgenomics Pers Med. 2017;10:69–78. 10.2147/PGPM.S107047 PMC5376125 28392709

